# Leisure activities as reserve mediators of the relationship between loneliness and cognition in aging

**DOI:** 10.1038/s41398-024-02960-6

**Published:** 2024-05-28

**Authors:** Chao Du, Xin Li, Jingyi Li, Wenxu Wang, Mingxi Dang, Jiayin Cheng, Kai Xu, Jun Wang, Chuansheng Chen, Yaojing Chen, Zhanjun Zhang

**Affiliations:** 1 State Key Laboratory of Cognitive Neuroscience and Learning, Beijing Normal University, Beijing, 100875 China; 2 Beijing Aging Brain Rejuvenation Initiative Centre, Beijing Normal University, Beijing, 100875 China; 3 Research Institute of Intelligent and Complex Systems, Fudan University, Shanghai, 200433 China; 4 CSSC System Engineering Research Institute, Beijing, 100036 China; 5 School of Systems Science, Beijing Normal University, Beijing, 100875 China; 6 Senior 2 Class 6, The High School Affiliated to Renmin University of China, Beijing, 100097 China; 7 School of Artificial Intelligence, Beijing Normal University, Beijing, 100875 China; 8 Department of Psychological Science, University of California, Irvine, CA 92697-7085 USA

**Keywords:** Human behaviour, Psychiatric disorders

## Abstract

Previous studies have found that loneliness affects cognitive functions in older persons. However, the influence of loneliness on different cognitive fields and the internal mechanism of the relationship are unclear. A total of 4772 older persons aged above 50 years (Mean = 65.31, SD = 6.96, 57.7% female) were included in this study. All the participants completed the characteristics scale, as well as the loneliness scale, leisure activity scale, and cognitive function tests in six domains. The results showed that 17.6% of participants had high loneliness, while 16.7% of participants had low loneliness. Associations were observed between higher levels of loneliness and lower scores in general cognitive ability, memory, and executive functions. Mediation analysis suggested that leisure activities, encompassing mental, physical, and social activities, were associated with cognitive functions in the context of loneliness. These results indicate that leisure activities may play a significant role in the relationship between loneliness and cognitive functions in older adults. The study highlights the importance of considering leisure activities in this demographic to potentially mitigate the adverse cognitive effects associated with loneliness.

## Introduction

Loneliness is defined as a subjective, unpleasant, and distressing phenomenon resulting from a discrepancy between an individual’s desired and achieved levels of social relations [1]. Loneliness is very common among older persons [1], and can cause a series of problems, including increased mortality [2], daytime dysfunction [3], and depression [4]. Moreover, some studies paid attention to the effect of loneliness on cognitive functions in the older persons and found that loneliness can cause the decline of various cognitive functions [5]. However, the cognitive domains affected by loneliness are different in these studies. For example, some studies found that loneliness was associated with memory [6], whereas others found that loneliness was associated with processing speed [5]. Recent studies have shown that interventions for loneliness can be varied and must be tailored to individual needs [7]. The diverse cognitive and social functions associated with loneliness are linked to different brain networks and structures, underscoring the importance of researching the differential impact of loneliness on cognitive subdomains [8]. Understanding the intricate relationship between loneliness and cognitive function highlights the necessity for targeted intervention measures. These interventions should be specifically designed to address the unique cognitive vulnerabilities linked to loneliness, thereby ensuring a more detailed and effective approach to this widespread issue. Therefore, more data is needed to clarify which cognitive areas loneliness primarily affects.

In recent years, some studies have explored the internal mechanism of loneliness affecting cognitive functions [9]. The change in leisure activity may be an important result caused by loneliness [10]. For example, studies have found that loneliness affects the willingness of older persons to participate in social activities [11] and physical activities [10], and these activities significantly affect the cognitive functions of older persons [12]. However, this mediating effect of leisure activities on the relationship between loneliness and cognition has not been explored.

Therefore, we used a large sample of data with a large age range to explore (1) which areas of cognitive functions does loneliness affect in the older persons; (2) whether leisure activities mediate the effect of loneliness on cognition in older persons.

## Methods

### Participants

This study included 10465 native Chinese participants from the Beijing Aging Brain Rejuvenation Initiative (BABRI), an ongoing longitudinal study examining the brain and cognitive decline in a community-dwelling sample of older persons. To ensure a representative sample, the cohort employed a multistage cluster sampling design. For a detailed description of the sampling methodology and cohort characteristics, readers are referred to the cohort introduction studies [13]. The participant inclusion criteria were as follows: (1) no less than 6 years of education; (2) aged between 50–85 years old, inclusive; (3) right-handed; (4) completed loneliness score and cognitive test score; (5) no less than 24 scores of mini-mental state examination (MMSE) [14]; (6) baseline data for each subject. All participants gave written informed consent to our protocol that was approved by the ethics committee of the State Key Laboratory of Cognitive Neuroscience and Learning, Beijing Normal University. Written consent was obtained from each subject. Finally, 4772 participants were included in the study.

### Neuropsychological testing

All participants underwent a series of neuropsychological assessments, including the MMSE, which is a brief cognitive screening tool evaluating orientation, memory, calculation, language, visuospatial abilities, and attention (scores range from 24–30 in this study). Additionally, they were tested in five cognitive domains using associated tests: (1) Memory: This was evaluated using the Auditory Verbal Learning Test (AVLT)-delay recall, which assesses memory through both immediate and delayed recall of 12 words over five trials, with the AVLT-delay being the fifth trial conducted 25 minutes after the initial test (score range from 0–12). Additionally, the Rey-Osterrieth Complex Figure (ROCF) test delay recall is utilized for assessing visuospatial abilities and memory, where ROFC-delay, a recall test conducted 20 minutes later, is scored out of 36 based on reproduction accuracy; (2) Language: This assessed using the Category Verbal Fluency Test (CVFT). This test challenges participants to list as many items as possible in categories like animals, fruits, and vegetables within one minute, with the total number of unique correct responses forming the test score. Furthermore, the Boston Naming Test (BNT) requires participants to name 30 simple line drawings, focusing on their ability to form verbal concepts, with a total score of 30; (3) Attention: This is evaluated using part A of the Trail Making Test (TMT-A), which involves connecting numbered and lettered circles in sequence, with the time taken indicating cognitive performance. Additionally, part B of the Stroop Color Word Test (SCWT-B) assesses attention by requiring participants to quickly and accurately read out colors presented in various sequences, with performance measured by response time; (4) Execution: This is assessed with part B of the Trail Making Test (TMT-B), which tasks participants with alternately connecting boxes with circles and squares in numerical order. Part C of the Stroop Color Word Test (SCWT-C) further assesses executive function by requiring participants to name the ink color of written words, contrasting with their textual meaning, with a focus on response speed; 5) Visual space: The Clock Drawing Test (CDT) assesses visuospatial abilities by asking participants to complete a clock face to indicate a specific time, with scoring based on the accuracy of the clock drawing process and clock face representation (score range from 0–30). The copy part of the ROCF (ROCF-copy) is also used to assess spatial construction skills (score range from 0–36). The specific neuropsychological test procedures have been described previously [15].

### Loneliness scale

Loneliness was assessed using the revised UCLA Loneliness Scale (UCLA), a widely used and reliable self-report measure [16]. The scale consists of 20 items probing satisfaction with social relationships in the past two weeks. An example item is, “How often do you feel that there is no one you can turn to?” Each item is rated on a 4-point Likert-type scale, where 1 = Never, 2 = Rarely, 3 = Sometimes, and 4 = Often. Total UCLA scores are calculated by summing items and reverse coding where necessary so that higher scores correspond to higher loneliness. Total scores can range from 20 to 80. The R-UCLA scale demonstrated high internal consistency in older (Cronbach’s α = 0.89). UCLA was treated as a continuous variable in this study.

### Leisure activity

Leisure activity was defined as activities in which individuals participated for enjoyment that was independent of work, including reading, writing, participating in senior citizen university, playing chess, poker, or mahjong and doing crafts, etc [17]. The scale consists of 23 items on involvement frequency with leisure activities, and each item is rated on a 5-point scale, where 1 = Never, 2 = More than once a year, 3 = More than once a month, 4 = More than once a week, 5 = Everyday. Leisure activities were classified into mental, physical, and social domains based on assigned weights from 0 to 3, reflecting their engagement levels. For instance, reading was allocated a weight of 3 for mental activity due to its cognitive demand, while receiving a 0 for physical and social activities, indicating minimal engagement in these areas. Conversely, playing chess was rated with a 3 in both mental and social domains for its intellectual stimulation and interactive nature, but a 0 in physical activity, demonstrating the absence of physical exertion. The score ranges for the three activities were as follows: for mental activity, 47 to 188; for physical activity, 37 to 148; and for social activity, 29 to 116.

### Statistical analysis

All statistical analyses were conducted using SPSS version 21.0. For each cognitive domain, scores were standardized by summing the standardized values of the two scales within that domain. To investigate the relationship between loneliness and cognitive function, we employed partial correlation, incorporating gender, age, education, marital status (whether married), employment status (whether retired), and income level (each level represents 500 CNY) as covariates to more effectively control for potential confounding variables. This comprehensive approach ensures that our results are robust, accounting for a wider range of sociodemographic influences on the observed relationships.

Furthermore, to examine how leisure activities mediate the relationship between loneliness and cognitive function, we utilized the Process plug-in in SPSS. This allowed us to test the indirect effects within our models using the Bootstrap method with 1000 resamples, while also controlling for gender, age, education, marital status, employment status, and income level. By including these additional covariates in both the partial correlation and mediation analyses, we enhance the validity of our findings, ensuring that they reflect the genuine effects of loneliness and leisure activities on cognitive function, free from the distortive influence of key sociodemographic factors.

## Results

### Descriptive statistics

The descriptive statistical results of demographic and cognitive variables are shown in Table 1. The average age of 4772 subjects in this study is 65.31 years (SD = 6.96), the average education level is 10.98 years (SD = 3.07), and 57.7% are women. The score distribution of loneliness is shown in Figure S1. The average score of loneliness was 34.09 (SD = 9.28). There were about 135 (2.8%) older persons with severe loneliness (UCLA score above 52), 707 (14.8%) older persons with moderate loneliness (52–44), and 212 (82.4%) older persons with mild or no loneliness (below 44). Also, the score ranges and distributions of various cognitive functions are shown in Figure S2.

**Table 1:** Demographics of participants (SDs or % in parentheses) (*n* = 4772).

Variable	Mean/Count
Age	65.31 (6.96)
Female	2753 (57.7%)
Education	10.98 (3.07)
Married	3974 (83.3%)
Retired	3652 (76.5%)
Income level	6.51 (2.92)
**Loneliness**	
UCLA	34.09 (9.28)
**Cognition**	
MMSE	27.58 (1.69)
**Memory**	
AVLT-delay	5.08 (2.58)
ROCF-delay	13.00 (6.49)
**Language**	
CVFT	43.84 (8.95)
BNT	22.53 (3.98)
**Attention**	
SCWT-B	40.86 (12.61)
TMT-A	60.86 (23.42)
**Execution**	
SCWT-C	82.06 (27.10)
TMT-B	168.74 (66.83)
**Visual space**	
ROCF-copy	33.37 (4.37)
CDT	23.66 (5.13)

*MMSE* Mini Mental State Examination, *AVLT* Auditory Verbal Learning Test, *ROCF* Rey–Osterrrieth Complex Figure, *CVFT* Category Verbal Fluency Test, *BNT* Boston Naming Test, *SCWT* Stroop Color Word Test, *TMT* Trail Making Test, *CDT* Clock-Drawing Test.

### The correlation between loneliness and cognition

In order to explore the relationship between loneliness and different cognitive domains, 11 cognitive scales were classified into MMSE and 5 specific cognitive domains, including memory, language, attention, execution, and visual space by standardizing, summing, and then re-standardizing scores within each domain. Partial correlation analyses revealed that loneliness was significantly and negatively correlated with several cognitive functions: MMSE (*r* = −0.029, *p* = 0.049), memory (*r* = −0.045, *p* = 0.002), and executive functions (*r* = −0.037, *p* = 0.012). This indicates that higher levels of loneliness are associated with diminished performance in these cognitive domains. Conversely, the analysis showed no significant correlations between loneliness and language (*r* = 0.012, *p* = 0.436), attention (*r* = −0.013, *p* = 0.372), or visual space (*r* = −0.004, *p* = 0.765), suggesting that loneliness does not significantly impact these specific cognitive functions (Table S1).

### The reserved role of leisure activities on the relationship between loneliness and cognition

**Fig. 1:**
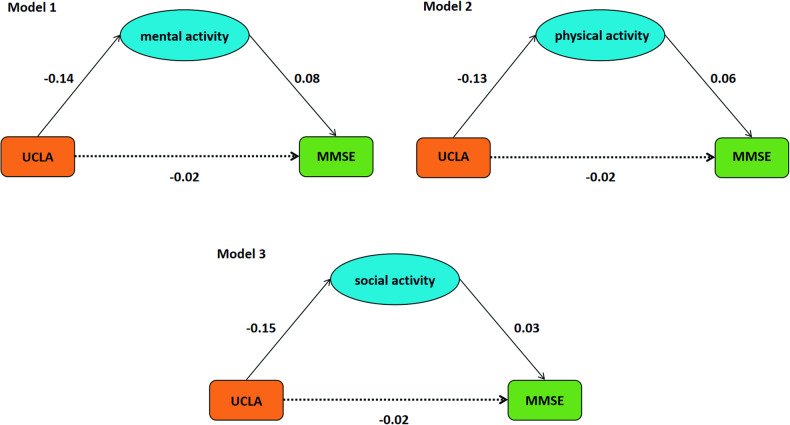
The models of loneliness and MMSE that mediated by mental, physical, and social activity, respectively. Standardized regression coefficients were shown on each path. The solid line represents a significant path, while the dotted line represents an insignificant path.

The nine models of leisure activities mediating the correlation between loneliness and cognitive functions (controlling for gender, age, education, marital status, employment status, and income level) are shown in Table 2 and Fig. 1 (taking MMSE as an example). The results of Bootstrap analyses showed that all the models of the indirect effects were significant, indicating the existence of mediation effects. Except for when the dependent variable is memory, the direct effects of physical and social activity are also significant, and the direct effects of all other mediation models are not significant. The results of the path coefficient showed that the higher the sense of loneliness, the less participation in leisure activities (mental activity, physical activity, and social activity), and the worse cognitive functions (MMSE, memory, and execution).

**Table 2:** Model parameters of mediation.

Model	Effect type	Estimate ± SE	95% CI
UCLA-mental activity-MMSE	Total	−0.0284 ± 0.0145	[−0.0567, −0.0001]
	Direct	−0.0170 ± 0.0146	[−0.0455, 0.0116]
	Indirect	−0.0114 ± 0.0023	[−0.0161, −0.0068]
UCLA-physical activity-MMSE	Total	−0.0284 ± 0.0145	[−0.0567, −0.0001]
	Direct	−0.0211 ± 0.0146	[−0.0496, 0.0075]
	Indirect	−0.0073 ± 0.0020	[−0.0115, −0.0037]
UCLA-social activity-MMSE	Total	−0.0284 ± 0.0145	[−0.0567, −0.0001]
	Direct	−0.0236 ± 0.0146	[−0.0523, 0.0050]
	Indirect	−0.0048 ± 0.0023	[−0.0095, −0.0003]
UCLA-mental activity-Memory	Total	−0.0439 ± 0.0144	[−0.0721, −0.0158]
	Direct	−0.0221 ± 0.0143	[−0.0502, 0.0060]
	Indirect	−0.0218 ± 0.0031	[−0.0283, −0.0160]
UCLA-physical activity-Memory	Total	−0.0439 ± 0.0144	[−0.0721, −0.0158]
	Direct	−0.0310 ± 0.0144	[−0.0593, −0.0027]
	Indirect	−0.0129 ± 0.0023	[−0.0177, −0.0085]
UCLA-social activity-Memory	Total	−0.0439 ± 0.0144	[−0.0721, −0.0158]
	Direct	−0.0331 ± 0.0145	[−0.0615, −0.0047]
	Indirect	−0.0108 ± 0.0024	[−0.0159, −0.0062]
UCLA-mental activity-Execution	Total	−0.0344 ± 0.0136	[−0.0611, −0.0077]
	Direct	−0.0169 ± 0.0137	[−0.0435, 0.0101]
	Indirect	−0.0175 ± 0.0026	[−0.0225, −0.0125]
UCLA-physical activity-Execution	Total	−0.0344 ± 0.0136	[−0.0611, −0.0077]
	Direct	−0.0249 ± 0.0137	[−0.0517, 0.0021]
	Indirect	−0.0095 ± 0.0021	[−0.0138, −0.0053]
UCLA-social activity-Execution	Total	−0.0344 ± 0.0136	[−0.0611, −0.0077]
	Direct	−0.0234 ± 0.0138	[−0.0503, 0.0036]
	Indirect	−0.0110 ± 0.0022	[−0.0157, −0.0071]

*SE* Standard Error, *CI* Confidence Interval, *MMSE* Mini Mental State Examination.

## Discussion

Here, we used a large sample to explore the relationships between loneliness and personal characteristics, as well as the effects of loneliness on various cognitive functions in the context of aging, and the mediating effect of leisure activities on this relationship. The related areas of cognition and loneliness are MMSE [18], executive function [19], and memory [6], which are consistent with previous studies. However, language, attention, and visual space are not related to loneliness in our sample. Two previous studies have found the same results about language and loneliness [18], as well as no study has reported a significant correlation between visual space and loneliness in aging. Unexpectedly, we did not find a relationship between loneliness and attention, which in some studies may be called processing speed [18], they found that significant and negative associations between loneliness and processing speed persisted even after controlling for a lot of additional factors. This discrepancy in findings may be attributed to our study’s focus on older Chinese adults, suggesting that cultural factors and distinct cognitive aging patterns in this demographic could influence the relationship between loneliness and specific cognitive functions, thereby highlighting the need for comparative cultural research to further explore these variations.

Recent studies reveal a complex relationship between loneliness and the default mode network (DMN) in the brain, showing not only a correlation between loneliness and alterations in the gray matter volume within the DMN but also significant associations with functional connectivity and white matter tracts in this network [20]. These findings suggest that loneliness has a profound impact on higher-order cognitive functions like self-referential thinking, memory retrieval, future planning, and emotion regulation, predominantly managed by the DMN. Additionally, loneliness is linked to heightened connectivity in the inferior frontal gyrus [21], which plays a crucial role in executive control. Importantly, while loneliness shows a significant correlation with the DMN, its impact on other cognitive domains such as attention, language, and spatial abilities appears to be less pronounced. These domains are predominantly modulated by other neural networks, including the attention network, language processing network, and visual-spatial network. This differential impact highlights the nuanced ways in which loneliness intersects with various cognitive functions, suggesting a more pronounced influence on introspective and self-relevant cognitive processes as opposed to externally directed cognitive functions. This aligns with other studies showing high loneliness associated with impaired executive control functioning, including cognitive subdomains like working memory, planning, response inhibition, and attention control [22]. This supports the hypothesis that loneliness, as an inward-oriented state reducing external interactions, disproportionately affects cognitive domains related to internal processing, such as memory and executive functions. Conversely, cognitive domains involving direct external engagement, like attention, language, and spatial abilities, modulated by different neural networks, appear less affected by loneliness. This differential impact underlines the nuanced ways loneliness intersects with various cognitive functions, suggesting a more pronounced influence on introspective and self-relevant cognitive processes.

The various leisure activities, including mental, physical, and social activities, maybe the mediating factors of the effects of loneliness on cognition. Previous studies have found that long-term loneliness of older persons may narrow their own social circle and reduce willingness to participate in various activities [10], whereas participation of these activities can significantly protect the cognitive functions of older persons [12]. It has been found that the changes in stress state [23], prolonged activation of the hypothalamus–pituitary–adrenal axis [24], and inflammatory reaction caused by leisure activities may be an important reason for the decline of cognitive functions [25].

This study suggested the importance of paying attention to the loneliness of older persons. Taking care of their mental health problems will not only help them to participate in leisure activities, and improve the quality of late life, but also help prevent dementia. In addition, families and communities should organize and encourage older people to participate in various leisure activities, including mental, physical, and social activities, to maintain their brain vitality and prevent cognitive decline. However, the limitation of this study is that it does not include neuroimaging or biomarkers to explore the impact of loneliness on cognitive functions. The inclusion of these indicators will better explain why loneliness affects part of cognition. Another limitation of our study is the reliance on cross-sectional observational data, which restricts our capacity to draw causal inferences. Although our analysis revealed statistically significant associations between loneliness, leisure activity participation, and cognitive function, these are correlational and should not be interpreted as causal. While our mediation analysis was informed by existing literature and theoretical frameworks suggesting directional relationships, the absence of longitudinal data precludes definitive conclusions about causality. Therefore, our findings should be viewed as highlighting potential relationships that merit further investigation, especially through longitudinal research, to establish causative links.

## Conclusions

This study identified the notable associations between higher levels of loneliness and poorer general cognitive ability, executive function, and memory in aging populations, with leisure activities including mental, physical, and social activities appearing to mediate these relationships. These findings highlight the importance of focusing on the mental health and daily activities of older adults, which may be beneficial in supporting cognitive health.

### Supplementary information

Supplementary material

Figure S1

Figure S2

## Data Availability

Data and code used in this study can be made available after a reasonable request to the authors following a formal data-sharing agreement.
