# Supplementary material for: Leisure activities as reserve mediators of the relationship between loneliness and cognition in aging
Source: Transl Psychiatry. 2024 May 28;14:217. doi: 10.1038/s41398-024-02960-6 (PMC11133303; doi:10.1038/s41398-024-02960-6)
Supplement: Supplementary file 1 — Supplementary material [file 41398_2024_2960_MOESM1_ESM.docx]

**Table S1** Partial correlation between loneliness and cognitive function (controlling for

gender, age, education, marital status, employment status, and income level)

| Variables | Partial r | p value |
| --- | --- | --- |
| MMSE | -0.029 | 0.049 |
| Memory | -0.045 | 0.002 |
| Language | 0.012 | 0.436 |
| Attention | -0.013 | 0.372 |
| Execution | -0.037 | 0.012 |
| Visual space | -0.004 | 0.765 |

MMSE, Mini Mental State Examination. For each cognitive domain, scores were standardized by summing the standardized values of the two scales within that domain.

**Figure S1** Density distribution of loneliness. Average loneliness is between the mean - 1SD and the mean + 1SD. Below average is between the mean -1SD and the mean - 1.96SD, which have reached the scale boundary. Above average is between the mean + 1SD and the mean + 1.96SD. Exceptional loneliness is above the mean + 1.96SD.

**Figure S2** Density distribution of various cognitive functions. MMSE, Mini Mental State Examination; AVLT.delay, delay recall of the Auditory Verbal Learning Test; ROCF.delay, delay recall of the Rey-Osterrieth Complex Figure; CVFT, the Category Verbal Fluency Test; BNT, the Boston Naming Test; SCWT.B, part B of the Stroop Color Word Test; TMT.A, part A of the Trail Making Test; SCWT.C, part C of the Stroop Color Word Test; TMT.B, part B of the Trail Making Test; ROCF.copy, copy part of the Rey-Osterrieth Complex Figure; CDT, the Clock Drawing Test.
